# The functional roles of the circRNA/Wnt axis in cancer

**DOI:** 10.1186/s12943-022-01582-0

**Published:** 2022-05-05

**Authors:** Chen Xue, Ganglei Li, Qiuxian Zheng, Xinyu Gu, Zhengyi Bao, Juan Lu, Lanjuan Li

**Affiliations:** 1 State Key Laboratory for Diagnosis and Treatment of Infectious Diseases, Collaborative Innovation Center for Diagnosis and Treatment of Infectious Diseases, The First Affiliated Hospital, College of Medicine, National Clinical Research Center for Infectious Diseases, Zhejiang University, No. 79 Qingchun Road, Shangcheng District, 310003 Hangzhou, China; 2 Department of Neurosurgery, The First Affiliated Hospital, College of Medicine, Zhejiang University, 310003 Hangzhou, China

**Keywords:** circRNA, Wnt, cancer, Biomarker, Mechanism

## Abstract

CircRNAs, covalently closed noncoding RNAs, are widely expressed in a wide range of species ranging from viruses to plants to mammals. CircRNAs were enriched in the Wnt pathway. Aberrant Wnt pathway activation is involved in the development of various types of cancers. Accumulating evidence indicates that the circRNA/Wnt axis modulates the expression of cancer-associated genes and then regulates cancer progression. Wnt pathway-related circRNA expression is obviously associated with many clinical characteristics. CircRNAs could regulate cell biological functions by interacting with the Wnt pathway. Moreover, Wnt pathway-related circRNAs are promising potential biomarkers for cancer diagnosis, prognosis evaluation, and treatment. In our review, we summarized the recent research progress on the role and clinical application of Wnt pathway-related circRNAs in tumorigenesis and progression.

## Background

Cancer is one of the main causes of death today and has become a serious public health problem worldwide [1–5]. It is a complex disease that involves changes in a variety of processes, including genetic and epigenetic characteristic changes [6–8]. The molecular changes in cancer genes and related signaling pathways could provide information for cancer diagnosis and targeted therapy [9–11]. This information could contribute to improvements in cancer diagnosis and treatment.

Human genome sequence data indicate that more than 98% of the genome is noncoding genes [12–14]. The transcripts of these genes lack protein-coding ability and are recognized as noncoding RNAs (ncRNAs) [15–18]. ncRNAs were once considered byproducts of transcription [19–21]. With the development of high-throughput sequencing technology, ncRNA features have gradually been revealed. ncRNAs comprise various types of RNA species, including microRNAs (miRNAs), long ncRNAs (lncRNAs), and circular RNAs (circRNAs) [22–24]. CircRNA is a single-stranded, covalently closed ncRNA without 5’ end caps or 3’ end poly (A) tails [25–28]. It is generated from its precursor mRNA by noncanonical splicing [29–31] and is widely expressed in a wide range of species ranging from viruses to plants to mammals [32, 33]. circRNAs may act as transcription modulators, miRNA sponges, or protein decoys to exert their function in cancer progression [34–36]. In addition, circRNAs are obviously associated with many clinical characteristics [37–41], which could provide important guidance for the accurate diagnosis and treatment of cancer. Accumulating evidence indicates that circRNAs play a pivotal role in the process of cancer and have the potential to be biomarkers in cancer diagnosis, prognosis, and treatment [42–46].

The Wnt pathway is an evolutionarily conserved pathway [47–49]. It plays a critical role in embryonic development, tissue renewal and regeneration [50–52]. The Wnt pathway can be divided into three classes: Wnt/β-catenin signaling, Wnt/planar cell polarity (PCP) signaling, and Wnt/Ca signaling [47, 53, 54]. Aberrant activation of the Wnt pathway is significantly correlated with a series of cancers, such as lung cancer [55–57], colorectal cancer [58, 59], bladder cancer [60, 61], osteosarcoma [62, 63], glioma [64, 65], and chronic lymphocytic leukemia [66, 67]. Accumulating evidence indicates that circRNAs regulate a series of cellular biological functions by interacting with the Wnt pathway in the cancer process [68–70]. These studies provided novel perspectives into cancer diagnosis and treatment. circRNAs related to the Wnt pathway have been the focus of many cancer research studies [63, 69, 71–73]. In this review, we summarized the recent research progress regarding the molecular mechanisms and functional roles of circRNAs related to the Wnt pathway in tumorigenesis and tumor progression.

### The wnt pathway in tumorigenesis

**Fig. 1:**
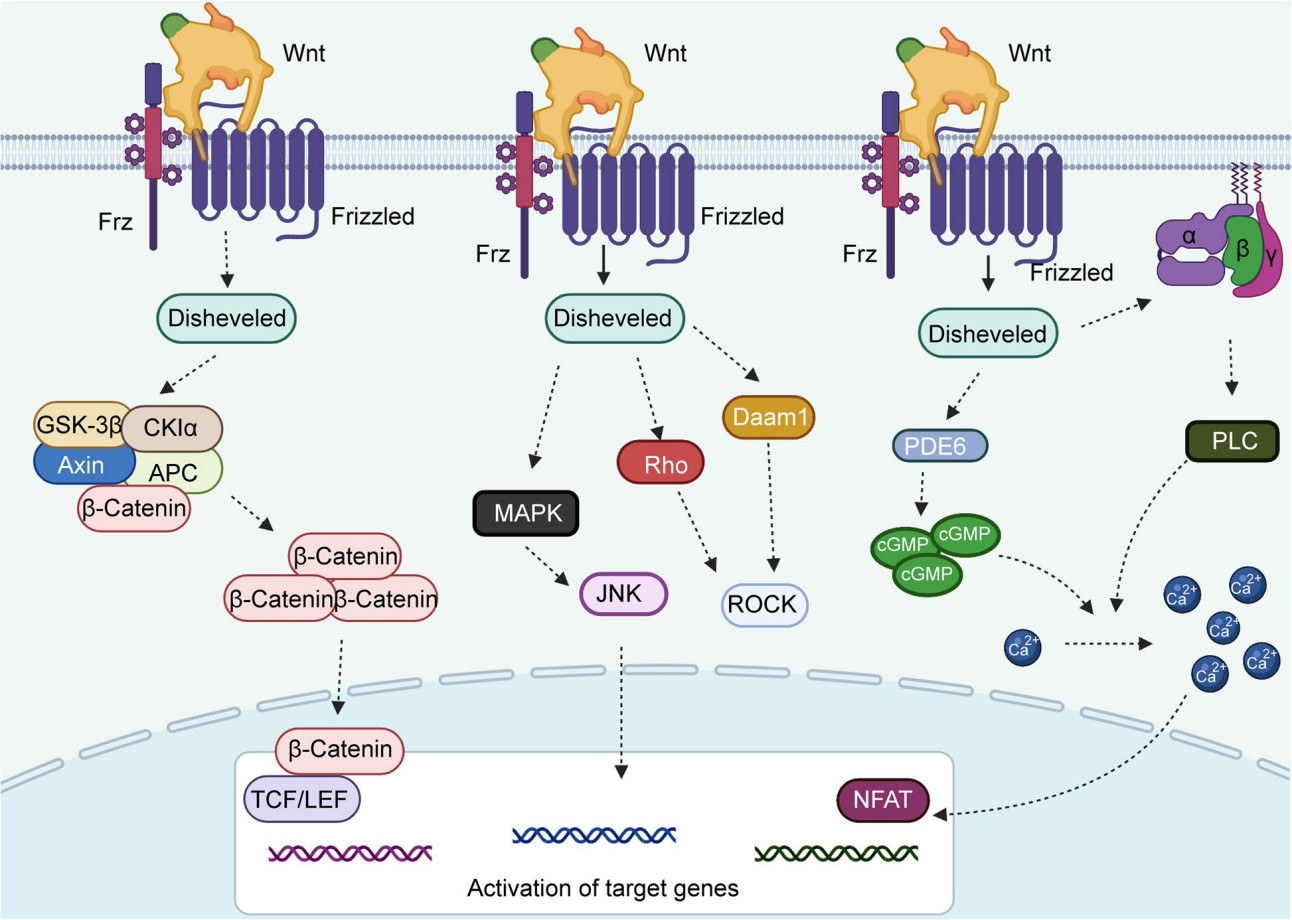
Wnts are a family of secreted, lipid-modified proteins that bind to Frizzled receptors to activate signaling cascades. The Wnt pathway can be divided into three classes: Wnt/β-catenin signaling, Wnt/PCP signaling, and Wnt/Ca signaling

The Wnt gene was first identified in mouse mammary tumors in 1982 [74–76]. At that time, it was designated as int1 [75, 77]. Because of the high homology between the mouse int1 gene and the Drosophila Wingless gene, the researchers merged Wingless with Int1 and assigned the name Wnt gene [78, 79]. The Wnt gene, localized at 12q13, mediates physiological effects in a paracrine and autocrine manner [78, 80]. The signaling pathways regulated by the Wnt gene are collectively termed the Wnt pathway. The Wnt signaling pathway is highly conserved from Drosophila to humans. The pathway [81–83] is critical for a wide variety of cellular functions, such as cell polarity, movement, proliferation, asymmetric division, and muscle tissue development. Wnts are a family of secreted, lipid-modified proteins that bind to Frizzled receptors to activate signaling cascades [84, 85]. The Wnt pathway can be divided into three classes: Wnt/β-catenin signaling, Wnt/planar cell polarity signaling, and Wnt/Ca signaling [86–89] (Fig. 1). Wnt/β-catenin signaling, a canonical Wnt signaling pathway, is involved in the regulation of gene expression [90–92]. Wnt/planar cell polarity signaling regulates cell polarity and directional cell movements [83, 93, 94]. Wnt/Ca signaling is obviously associated with the release of intracellular calcium [95, 96]. Dysregulation of the Wnt pathway has a strong relevance to cancer.

### Wnt/β-catenin signaling

The Wnt/β-catenin signaling pathway is characterized by the cellular redistribution and nuclear accumulation of the β-catenin gene [97, 98]. Wnt protein combines with Frz and LRP5/6 on the cell surface to form a trimer, which transmits the signal and activates the protein Disheveled [Dsh/DVL] [99, 100]. This leads to the disassociation of the β-catenin degradation complex adenomatous polyposis coli (APC)/Axin/GSK-3β (glycogen synthase kinase 3β) and increases the cytoplasmic levels of β-catenin [101, 102]. Then, upregulated β-catenin is transferred into the nucleus. Nuclear β-catenin interacts with T cell transcription factor (TCF])/lymphoid enhancer factor (LEF) and finally activates the expression of downstream target genes [98, 103–105]. The Wnt/β-catenin signaling pathway participates in the cancer process by acting as an important modulator [106–108] of cell proliferation, metastasis, and differentiation. Overexpression of the Wnt gene or mutation in one of the components that causes β-catenin degradation leads to activation of the Wnt/β-catenin pathway.

### Wnt/PCP signaling

In the Wnt/PCP signaling pathway, Wnt binds to frizzled transmembrane receptors and then activates the protein Disheveled (Dsh/DVL), leading to a series of cell signaling cascades [109–112]. DSH is connected to the downstream effectors Rho and ROCK (Rho-associated kinase) through Daam1. RAC is directly activated by Dsh, and Dsh further activates JNK by activating mitogen-activated protein kinases (MAP3Ks) and MAP2Ks [113, 114]. The PCP pathway is associated with cell polarity, cell alignment and cell migration.

### Wnt/Ca 2 + signaling

In the Wnt/Ca 2 + pathway, the Wnt protein is mainly composed of Wnt1, Wnt5A and Wnt11 and binds to the Frizzled transmembrane receptor on the cell surface [115, 116]. The combination of the Wnt protein and Frizzled activates Disheveled, which activates PLC through the G protein [117, 118]. These cellular processes could finally promote the release of intracellular Ca2+. The activation of Disheveled could also activate the cGMP-specific phosphodiesterase PDE6 and reduce intracellular cGMP, which leads to an increase in the intracellular Ca2 + concentration [119–123]. Elevated cytoplasmic Ca2 + concentrations can stimulate the nuclear factor NFAT and other transcription factors [124, 125]. These processes trigger the activation of downstream pathways and a series of altered cell functions. The Wnt/Ca2 + pathway is essential for early embryonic development, interneural communication and the inflammatory response [126, 127].

### CircRNA in the wnt pathway

CircRNAs, first found in the 1890s [128], remain enigmatic owing to technological limitations and limited existing knowledge. In 2013, Hansen TB et al. first proposed and confirmed that circRNAs function as miRNA sponges [129]. This finding started a new era in circRNA research [32, 42, 130–133]. Unlike linear RNAs, circRNAs are single-stranded, covalently closed noncoding RNAs without 5’ end caps or 3’ end poly (A) tails [25–28]. CircRNAs are not affected by RNA exonuclease, and their expression is more stable [134, 135]. CircRNAs are formed by reverse splicing events [29–31]. A mechanistic model argued that the RNA is partially folded during the transcription of pre-RNA. Initially, nonadjacent exons are pulled closer by RNA folding, and exon skipping occurs. The spanned region forms a circular RNA intermediate, and then circRNAs are formed by further splicing. Another model suggests that the reverse complement sequence located in the intron region causes the intron region to pair and mediate reverse splicing to form circRNA [136–140].

CircRNAs act mainly through four molecular mechanisms. In regulating gene expression, circRNAs affect the expression of parental gene mRNA by interacting with RNA binding proteins [141–143]. Competitive complementary pairing between introns can strike a balance with linear RNAs during the formation of circRNAs. CircRNAs can also exert their functions by acting as competing endogenous RNAs (ceRNAs) of miRNAs [144–147]. In addition, circRNAs are involved in the immune response [29, 148, 149]. Endogenous circRNAs play a role in the antiviral response, while exogenous circRNAs can stimulate immune signaling in mammalian cells by activating the pattern recognition receptor RIG-L [150–153]. Moreover, although circRNAs are noncoding RNAs, a few circRNAs can also perform regulatory functions by encoding peptides [154–156]. Several previous studies have shown that circRNAs play an important role in tumorigenesis and tumor progression. CircRNA_403658 facilitates aerobic glycolysis and cell growth by upregulating LDHA expression in bladder cancer [157]. CircRNA_103809 functions as an oncogene in the progression of hepatocellular carcinoma [158].

Both circRNAs and the Wnt pathway play a critical role in cancer development and progression. CircRNAs negatively or positively regulate cancer initiation, promotion, and progression by directly or indirectly interacting with the Wnt pathway. The interaction of circRNAs and the Wnt pathway has a noticeable impact on cell growth, metastasis, and other malignant cell behaviors in cancer. The majority of circRNAs act as sponges of miRNAs to activate or inactivate the Wnt pathway. With the deepening of research, more action modes between circRNAs and the Wnt pathway will be found. Related studies are expected to provide new insights for the diagnosis and treatment of cancer.

### The role of the circRNA/Wnt axis in cancer

CircRNAs related to the Wnt pathway are aberrantly expressed in many cancers. Emerging evidence suggests that a range of clinical characteristics have been associated with circRNAs related to the Wnt pathway (Table 1). Moreover, the circRNA/Wnt axis contributes to cancer progression by modulating many cell biological functions. In this section, we will introduce the expression, corresponding clinical features, functions and mechanisms of the circRNAs/Wnt axis (Table 2).

**Table 1:** Expression and characteristic features of cancer-related circRNAs in the Wnt pathway

Type	circRNA	Expression	Prognostic indicator	Clinical feature	Refs
Gastric cancer	circ0005654	Upregulated	Overall survival		[159]
Gastric cancer	circ_SMAD4	Upregulated	Overall survival		[73]
Gastric cancer	cir-ITCH	Downregulated	Overall survival	Lymph node metastasis	[160]
Gastric cancer	circHIPK3	Upregulated	Overall survival		[161]
Colorectal cancer	circRASSF2	Upregulated	Overall survival		[162]
Colorectal cancer	circ_0082182	Upregulated	Overall survival	TNM stage	[163]
Colorectal cancer	circ-PRKDC	Upregulated		TNM grades, lymph node metastasis, tumor size, and 5-FU Resistance	[164]
Colorectal cancer	circ5615	Upregulated	Overall survival	T stage	[165]
Colorectal cancer	circCCT3	Upregulated	Disease-free survival	Advanced stage	[166]
Colorectal cancer	circ _ 0005075	Upregulated	Overall survival, and disease-free survival	Histology/differentiation, invasion depth, and TNM stage	[167]
Colorectal cancer	circMTO1	Downregulated	Overall survival	Advanced TNM stage, and lymph node metastasis	[70]
Colorectal cancer	circRNA_100290	Upregulated	Overall survival	Tumor metastasis	[168]
Liver cancer	circ_0004018	Downregulated		Tumor size	[169]
Liver cancer	circ_0003418	Downregulated		Tumor size, TNM stage, and HBsAg level	[170]
Liver cancer	circZKSCAN1		Overall, and recurrence-free survival rate		[171]
Liver cancer	circZFR	Upregulated	Overall survival		[172]
Liver cancer	circ_0067934	Upregulated	Overall survival	TNM stage	[173]
Liver cancer	circ-ITCH	Downregulated	Overall survival		[174]
Lung cancer	circ_000984	Upregulated	Overall survival, and disease-free survival	TNM stage, and lymph nodes metastasis	[175]
Lung cancer	circ_001569	Upregulated	Overall survival	Tumor differentiation, lymph node metastasis, and TNM stage	[176]
Lung cancer	circ_0001946	Upregulated	Overall survival	TNM stage, and tumor size	[177]
Lung cancer	circ_0018414	Downregulated	Overall survival		[178]
Lung cancer	circ_0006427	Downregulated	Overall survival		[179]
Lung cancer	circ_0007059	Downregulated		Different stages	[180]
Lung cancer	cir-ITCH	Downregulated		Age	[181]
Glioma	circ_0001730	Upregulated		Clinical stage	[182]
Glioma	circ_0000177	Upregulated	Overall survival		[183]
Ovarian cancer	circPLEKHM3	Downregulated	Overall survival, and recurrence-free survival		[184]
Endometrial carcinoma	circ_0109046	Upregulated	5-year survival		[185]
Endometrial carcinoma	circ_0002577	Upregulated	Overall survival rate	FIGO stage, and lymph node metastasis	[186]
Osteosarcoma	circ_0002052	Downregulated	Overall survival, and progression-free survival		[187]
Thyroid cancer	circ-ITCH	Downregulated		Clinical stage, and lymph node metastasis	[188]
Breast cancer	circ-EIF6	Upregulated	Overall survival	Histological grade, and distant metastasis	[189]
Breast cancer	circ-ITCH	Downregulated	Overall survival		[190]
Breast cancer	circRNA_069718	Upregulated	Overall survival	TNM stage, and lymph node metastasis	[191]

**Table 2:** The functions and mechanisms of Wnt-associated circRNAs in cancer

Category	Type	circRNA	Role	Functions	Targeted molecule	Refs
Digestive tumors	Esophageal cancer	circRNA_100367	Oncogene	EMT, proliferation, migration, and radioresistance	miR-217, and Wnt3	[192]
	Esophageal cancer	cir-ITCH	Tumor suppressor	Cell cycle, and cell proliferation	miRNA, Wnt	[193]
	Gastric cancer	circ0005654	Oncogene	Proliferation, migration, and invasiveness	miR-363, *SP1*, Wnt, and β-catenin	[159]
	Gastric cancer	circRNA _ asap2	Oncogene	Proliferation, migration, invasion, and cell apoptosis	Wnt, and β-catenin	[194]
	Gastric cancer	circ-SFMBT2	Oncogene	Proliferation, migration, invasion, cell apoptosis, and oxidative stress	miR-885-3p, *CHD7*, Wnt, and β-catenin	[195]
	Gastric cancer	circCNIH4	Tumor suppressor	Proliferation, migration, invasion, and cell apoptosis	*DKK2*. *FRZB*, Wnt, and β-catenin	[196]
	Gastric cancer	circ_SMAD4	Oncogene	Proliferation, and cell apoptosis	miR-1276, *CTNNB1*, Wnt, and β-catenin	[73]
	Gastric cancer	circRNA_0044516	Oncogene	Proliferation, and cell apoptosis	miR-149, Wnt1, and β-catenin	[197]
	Gastric cancer	cir-ITCH	Tumor suppressor	Cell proliferation, migration, and invasion	miR-17, Wnt, and β-catenin	[160]
	Gastric cancer	circ_0001649	Tumor suppressor	Proliferation, migration, invasion, and cell apoptosis	miR-20a, *ERK*, and Wnt/β-catenin	[198]
	Gastric cancer	circHIPK3	Oncogene	Proliferation, and migration	Wnt1, and β-catenin	[161]
	Colorectal cancer	circ_0038718	Oncogene	Cell proliferation, migration, and invasion	miR-195-5p, *Axin2*, and Wnt/β-catenin	[199]
	Colorectal cancer	circ_0026628	Oncogene	Cell proliferation, migration, and stemness	miR-346, FUS protein, *SP1*, Wnt/β-catenin, and Sox2	[200]
	Colorectal tumor	cis-HOX	Oncogene	Self-renewal, tumorigenesis, and metastatic capacities of TICs	*FZD3*, Wnt/β-catenin, and KSRP	[201]
	Colorectal cancer	circRASSF2	Oncogene	Cell proliferation, migration, invasion, and cell apoptosis	miR-195-5p, *FZD4*, Wnt, and β-catenin	[162]
	Colorectal cancer	circSMARCA5	Tumor suppressor	Cell proliferation, migration, and invasion	miR-552, Wnt, and *YAP1*	[202]
	Colorectal cancer	circ_0082182	Oncogene	Cell proliferation, cell cycle, apoptosis, and metastasis	miR-411, miR-1205, and Wnt/β-catenin	[163]
	Colorectal cancer	circAGFG1	Oncogene	Cell proliferation, migration, stemness, and apoptosis	mir-4262, miR-185-5p, *YY1*, *CTNNB1*, Wnt, and β-catenin	[203]
	Colorectal cancer	circ-PRKDC	oncogene	5-FU resistance, cell proliferation, and invasion	*FOXM1*, miR-375, Wnt, and β-catenin	[164]
	Colorectal cancer	circ5615	Oncogene	Cell proliferation, cell cycle, and invasion	miR-149-5p, *TNKS*, Wnt, and β-catenin	[165]
	Colorectal cancer	circ-ABCC1	Oncogene	Cell stemness, sphere formation, and metastasis	Wnt	[204]
	Colorectal cancer	circCCT3	Oncogene	Cell invasion, and apoptosis	miR-613, Wnt3, miR-613, and *VEGFA*	[166]
	Colorectal cancer	circ _ 0009361	Tumor suppressor	Cell proliferation, migration, invasion, and EMT	miR-582, Wnt, and β-catenin	[205]
	Colorectal cancer	circ _ 0005075	Oncogene	Cell proliferation, migration, and invasion	Wnt, and β-catenin	[167]
	Colorectal cancer	circMTO1	Tumor suppressor	Cell proliferation, and invasion	Wnt, and β-catenin	[70]
	Colorectal cancer	circ _ 0000523	Tumor suppressor	Cell proliferation, and apoptosis	miR-31, Wnt, and β-catenin	[206]
	Colorectal cancer	circRNA_100290	Oncogene	Cell proliferation, migration, and invasion	miR-516b, *FZD4*, Wnt, and β-catenin	[168]
	Colorectal cancer	cir-ITCH	Tumor suppressor	Cell proliferation	Wnt, and β-catenin	[207]
	Liver cancer	circRNA-SORE	Oncogene	Sorafenib resistance, and apoptosis.,	Wnt, and β-catenin	[71]
	Liver cancer	circ_0004018	Tumor suppressor	Cell proliferation, and migration	miR-626, *DKK3*, Wnt, and β-catenin	[169]
	Liver cancer	circ-ITCH	Tumor suppressor	Cell proliferation, and apoptosis	Wnt, β-catenin, *c-Myc*, and CyclinD1	[174, 208]
	Liver cancer	circ_0003418	Tumor suppressor	Cell proliferation, migration, invasion, and cisplatin resistance	Wnt, and β-catenin	[170]
	Liver cancer	circZKSCAN1	Tumor suppressor	Cell stemness	*FMRP*, *CCAR1*, and Wnt	[171]
	Liver cancer	cZNF292	Oncogene	Cell proliferation, and cell cycle	Wnt, β-catenin, and *SOX9*	[209]
	Liver cancer	circβ-catenin	Oncogene	Cell growth, cell cycle, migration, and invasion	Wnt, and β-catenin	[210]
	Liver cancer	circZFR	Oncogene	Cell proliferation and EMT	Wnt, and β-catenin	[172]
	Liver cancer	circ_0067934	Oncogene	Cell proliferation, migration, invasion, and apoptosis	miR-1324, *FZD5*, and β-catenin	[173]
	Liver cancer	circ-ITCH	Tumor suppressor			[174]
	Pancreatic cancer	circ_0030167	Tumor suppressor	Cell invasion, migration, proliferation and stemness	miR-338-5p, *Wip1*, Wnt 8, and β-catenin	[211]
Respiratory system tumors	Lung cancer	circ-PGC	Oncogene	Cell viability, colony formation, cell migration, invasion, and glycolysis metabolism	miR-532-3p, *FOXR2*, Wnt, and β-catenin	[212]
	Lung cancer	circ-ZNF124	Oncogene	Cell proliferation, invasion, apoptosis, and cycle arrest	miR-498, *YES*, Wnt, and β-catenin	[213]
	Lung cancer	circ-BIRC6	Oncogene	Cell proliferation, migration and invasion, and apoptosis	miR-4491, Wnt2B, and β-catenin	[214]
	Lung cancer	circ_0067934	Oncogene	Cell proliferation, migration, invasion, and apoptosis	miR-1182, *Klf8*, Wnt, and β-catenin	[215]
	Lung cancer	circ_000984	Oncogene	Cell proliferation, migration, invasion, and EMT	Wnt, and β-catenin	[175]
	Lung cancer	circ_001569	Oncogene	Cell proliferation	Wnt, and β-catenin	[176]
	Lung cancer	circ_0043256	Oncogene	Cell proliferation, and apoptosis	miR-1252, *ITCH*, and Wnt	[216]
	Lung cancer	circ-SOX4	Oncogene	Cell proliferation, invasion, and migration	miR-1270, *PLAGL2*, and Wnt	[69]
	Lung cancer	circ_0001946	Oncogene	Cell proliferation	miR-135a-5p, *SIRT1*, Wnt, and β-catenin	[177]
	Lung cancer	circ_0018414	Tumor suppressor	Cell proliferation, stemness, and apoptosis	miR-6807-3p, *DKK1*, Wnt, and β-catenin	[178]
	Lung cancer	circ_0006427	Tumor suppressor	Cell proliferation, migration, and invasion	miR-6783-3p, *DKK1*, Wnt, and β-catenin	[179]
	Lung cancer	circ_0007059	Tumor suppressor	Cell proliferation, apoptosis, and EMT	miR-378, Wnt, and β-catenin	[180]
	Lung cancer	cir-ITCH	Tumor suppressor	Cell proliferation	Wnt, and β-catenin	[181]
Nervous system neoplasms	Glioma	circ_0001730	Oncogene	Cell proliferation, and migration	*Sp1*, miR-326, Wnt7B, and β-catenin	[182]
	Glioma	circKIF4A	Oncogene	Colony formation, migration, invasion, and apoptosis	miR-139-3p, Wnt, and β-catenin	[217]
	Glioma	circ_0000177	Oncogene	Cell proliferation, and invasion	miR-638, *FZD7*, and Wnt	[183]
	Glioma	cZNF292	Oncogene	Cell proliferation, cell cycle, and angiogenic potential	Wnt, and β-catenin	[218]
Genitourinary tumors	Prostate cancer	cir-ITCH	Tumor suppressor	Cell viability, and invasion	miR-17, Wnt, β-Catenin, PI3K, AKT, and mTOR	[219]
	Ovarian cancer	circABCB10	Oncogene	Cell proliferation, invasion, and cell apoptosis	miR-1271, *Capn4*, Wnt, and βcatenin	[220]
	Ovarian cancer	circPLEKHM3	Tumor suppressor	Cell growth, and migration	miR-9, *BRCA1*, *DNAJB6*, *KLF4*, *Akt1*, Wnt, and β-catenin	[184]
	Endometrial carcinoma	circ_0109046	Oncogene	Cell proliferation, aggressiveness, and apoptosis	miR105, *SOX9*, Wnt, and β-catenin	[185]
	Endometrial carcinoma	circ_0002577	Oncogene	Cell proliferation, migration, and invasion	miR-197, *CTNND1*, Wnt, and β-catenin	[186]
	Cervical cancer	circSAMD11	Oncogene	Cell proliferation, migration, invasion, and apoptosis	miR-503, *SOX4*, Wnt, and β-catenin	[221]
Blood system cancers	Acute myeloid leukemia	circ_0121582	Tumor suppressor	Cell proliferation, and cell cycle	miR-224, *GSK3β*, Wnt, and β-catenin	[222]
	Chronic lymphocytic leukemia	circ-CBFB	Oncogene	Cell proliferation, cell cycle, and apoptosis	miR-607, *FZD3*, Wnt, and β-catenin	[223]
	Diffuse large B-cell lymphoma	circ-APC	Tumor suppressor	Cell viability, and cell cycle	Wnt, β-catenin, *TET1*, and miR-888	[224]
Musculoskeletal system tumors	Osteosarcoma	circUBAP2	Oncogene	Cell proliferation, migration, invasion, apoptosis, and cisplatin resistance	miR-506-3p, *SEMA6D*, Wnt, and β-catenin	[225]
	Osteosarcoma	circMYO10	Oncogene	Cell proliferation, and emt	miR-370-3p, *RUVBL1*, Wnt, and β-catenin	[63]
	Osteosarcoma	circ_0002052	Tumor suppressor	Cell proliferation, migration, invasion, and apoptosis	miR1205, *APC2*, Wnt, and β-catenin	[187]
Endocrine system tumors	Thyroid cancer	circRNA_102171	Oncogene	Cell proliferation, migration, invasion, and apoptosis.	*CTNNBIP1*, β-catenin, *TCF3*, *TCF4*, *LEF1* complex, Wnt, and β-catenin	[226]
	Thyroid cancer	circRNA_NEK6	Oncogene	Cell growth, and invasion	*FZD8*, Wnt, and miR-370-3p	[227]
	Thyroid cancer	circ-ITCH	Tumor suppressor	Cell proliferation, invasion, and apoptosis	miR-22-3p, *CBL*, and β-catenin	[188]
Other systems tumors	Breast cancer	circ-EIF6	Oncogene	Cell proliferation, migration, and invasion	*MYH9*, Wnt, β-catenin, and *EIF6-224aa*	[189]
	Breast cancer	circARL8B	Oncogene	Cell viability, migration, invasion, and fatty acid metabolism	miR-653-5p, *PGE2*, PI3K, AKT, *GSK-3β*, Wnt, and β-catenin	[228]
	Breast cancer	circABCC4	Oncogene	Cell viability, migration, invasion, and apoptosis	miR-154-5p, *NF-κB*, Wnt, and β-catenin	[229]
	Breast cancer	circ-ITCH	Tumor suppressor	Cell proliferation, invasion, and metastasis	miR-214, miR-17, *ITCH*, Wnt, and β-catenin	[190]
	Breast cancer	circRNA_069718	Oncogene	Cell proliferation, and invasion	Wnt, and β-catenin	[191]
	Breast cancer	circFAT1	Oncogene	Cell apoptosis, migration, invasion, and oxaliplatin resistance	miR-525-5p, *SKA1*, *Notch*, and Wnt	[230]
	Melanoma	circ_0119872	Oncogene	Cell proliferation, and angiogenesis	miR-622, *G3BP1*, Wnt, β-catenin, and mTOR	[231]
	Melanoma	circ-GLI1 (circ_0027247)	Oncogene	Cell metastasis, and angiogenesis	*p70S6K2*, *Hedgehog*, *GLI1*, *Cyr61*, Wnt, and β-catenin	[232]
	Melanoma	circ_0084043	Oncogene	Cell proliferation, migration, invasion, and apoptosis	miR-429, and *homolog 2*, Wnt, and β-catenin	[233]

### Digestive tumors

#### Esophageal cancer

**Fig. 2:**
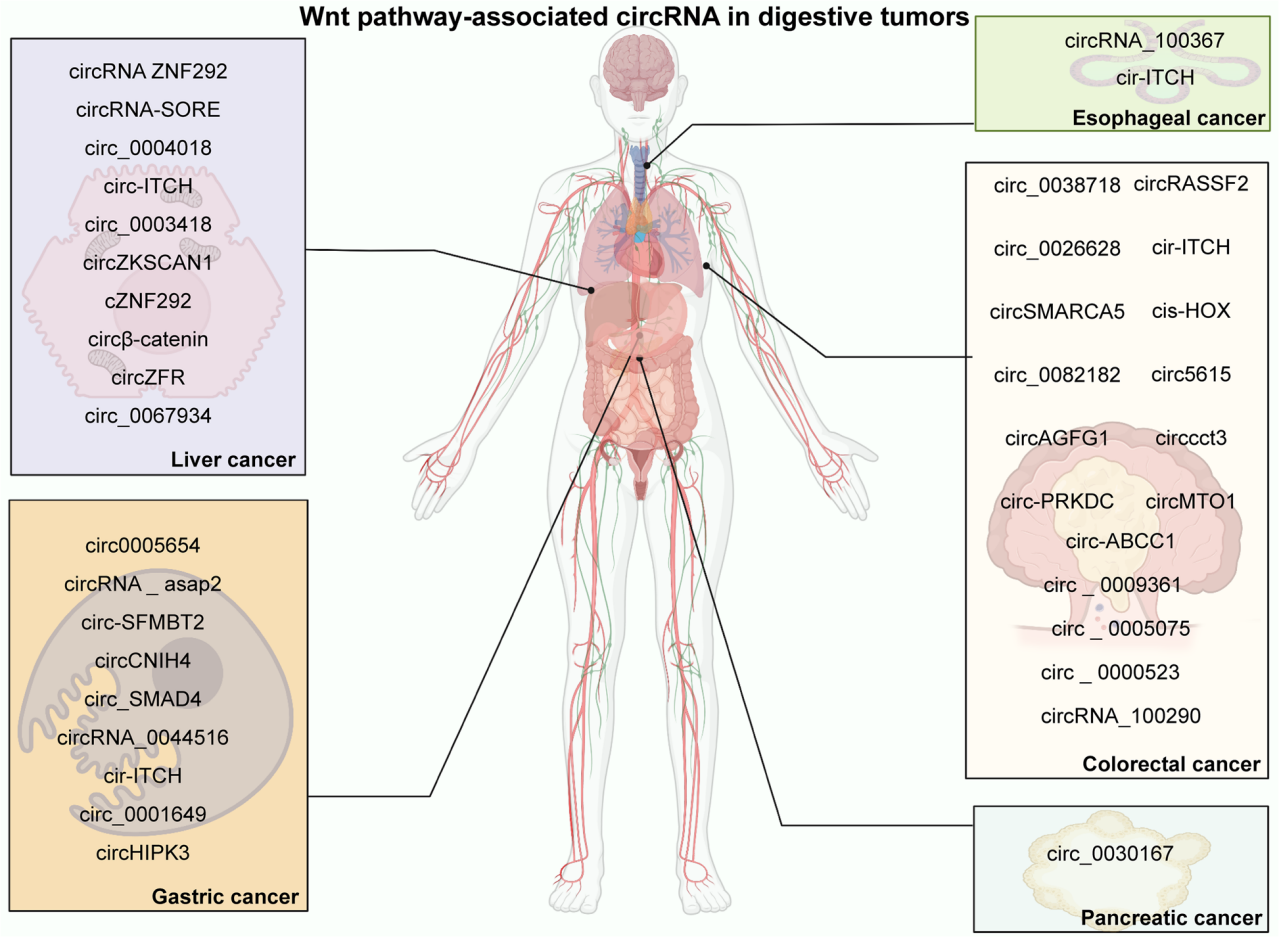
Wnt pathway-associated circRNAs in digestive tumors

Elevated levels of circRNA_100367 were observed in radioresistant esophageal cancer cell lines [192], while the expression of cir-ITCH was downregulated in esophageal squamous cell carcinoma (ESCC) tissues [193] (Fig. 2). The expression of cir-ITCH is positively associated with linear ITCH in ESCC. Functionally, colony formation and Cell Counting Kit-8 (CCK-8) assays showed that cir-ITCH could inhibit ESCC tumor growth through the regulation of cell proliferation. Knockdown of circRNA_100367 attenuates cell proliferation, migration, and radioresistance in esophageal cancer [192]. circRNA_100367 decreases radiation sensitivity by regulating the miR-217/Wnt3 pathway. CircRNA_100367 could also affect esophageal cancer cell growth under irradiation *in vivo*. Using bioinformatics tools, Su et al. [234] found that a large number of circRNAs were closely related to cancer progression. Further studies on these molecules are still required.

#### Gastric cancer

Some Wnt pathway-related circRNAs (circ0005654, circ-SFMBT2, circ_SMAD4, circRNA_0044516, and circHIPK3) are markedly upregulated in gastric cancer [73, 159, 161, 195, 197]. The expression of circ0005654, circ_SMAD4, circHIPK3, and circheckd1 are positively associated with a poor prognosis in patients with gastric cancer [73, 159–161]. Functionally, these circRNAs all contribute to promoting tumor cell proliferation in gastric cancer [73, 159, 161, 194, 195, 197]. Additionally, circ0005654, circRNA_ASAP2, circ-SFMBT2, and circHIPK3 obviously promote gastric cancer cell migration and invasion. Circ-SFMBT2 upregulation indicates higher levels of oxidative stress in gastric cancer [195]. Mechanistically, *in vitro* and *in vivo* studies demonstrated that circ0005654 functions as a ceRNA of miR-363 to upregulate sp1 in the process of gastric cancer [159]. The level of CTNNB1 is regulated by circ-SFMBT2, a sponge of miR-1276 [73]. Circ-SFMBT2 activates the Wnt/β-catenin pathway by upregulating CTNNB1 expression. CircRNA_0044516 affects cancer progression by regulating the miR-149/Wnt1/β-catenin axis [197].

**Fig. 3:**
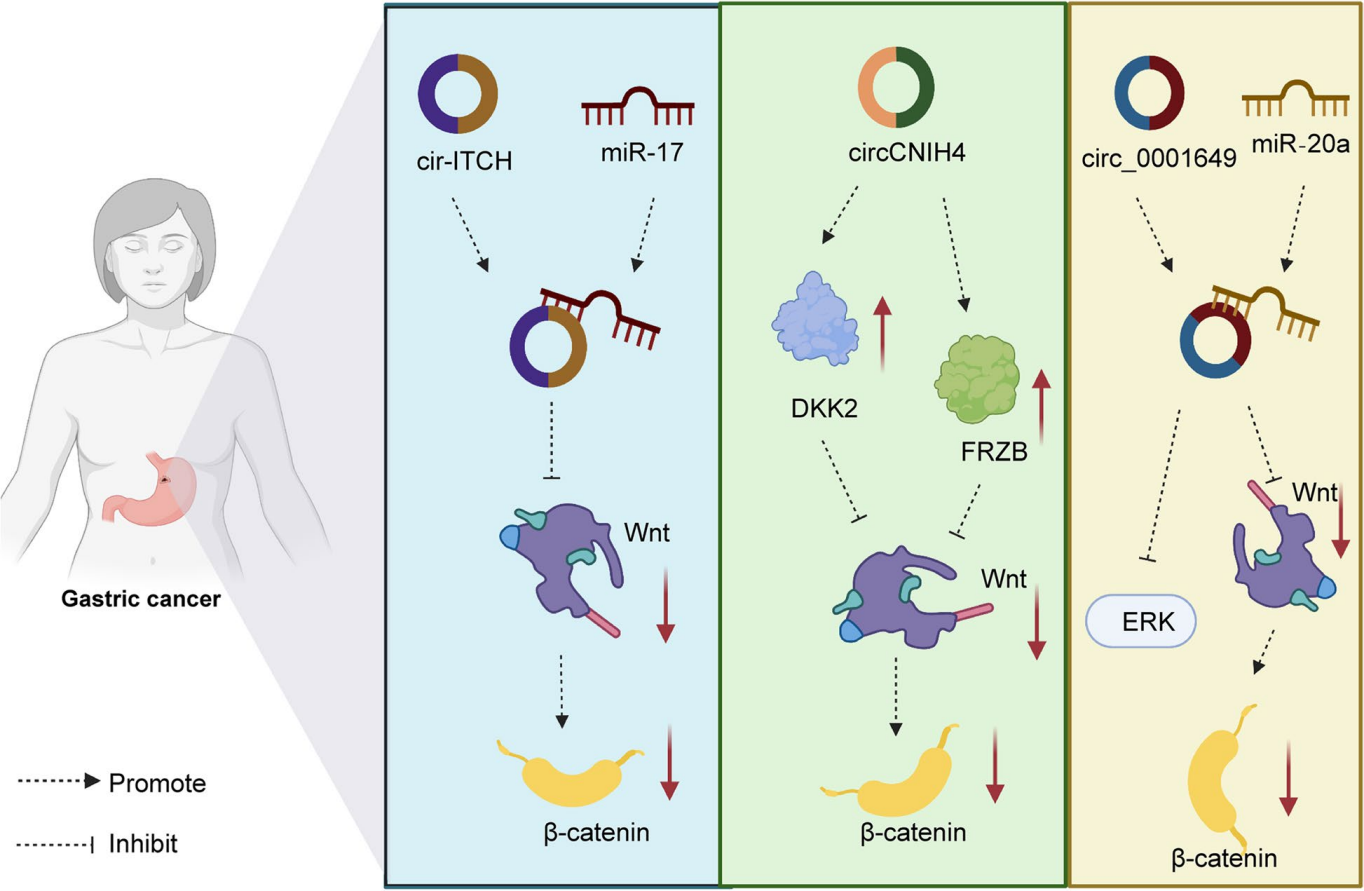
The mechanisms of Wnt-associated circRNAs in gastric cancer. **A** Cir-ITCH downregulates miR-17 expression to inactivate the Wnt/β-catenin pathway. **B** CircCNIH4 inhibits the Wnt/β-catenin pathway by upregulating DKK2 and FRZB levels in gastric cancer. **C** Circ_0001649 inhibits the ERK and Wnt/β-catenin signaling pathways by acting as a sponge of miR-20a in gastric cancer

Interestingly, some researchers found that the expression of circCNIH4, cir-ITCH, and circ_0001649 was significantly downregulated in gastric cancer tissues and cells [160, 196, 198]. cir-ITCH is closely related to lymph node metastasis and patient prognosis [160]. CircCNIH4, cir-ITCH, and circ_0001649 markedly reduced cell proliferation, migration, and invasion in gastric cancer cell lines. CircCNIH4 and circ_0001649 also contribute to gastric cancer progression through the regulation of cell apoptosis [196, 198]. CircCNIH4 inhibits the Wnt/β-catenin pathway by upregulating DKK2 and FRZB levels (Fig. 3). Similarly, cir-ITCH reduce miR-17 levels to inactivate the Wnt/β-catenin pathway. Circ_0001649 inhibits the ERK and Wnt/β-catenin signaling pathways by sponging miR‐20a.

#### Colorectal cancer [CRC]

CircRNA dysregulation has been discovered to be closely related to the occurrence and progression of CRC. Wnt pathway-associated circRNAs of CRC are shown in Table 1 [70, 162–168, 199–207]. Circ_0082182, circ-PRKDC, circ5615, and circ_0005075 are significantly correlated with advanced tumor-node-metastasis (TNM) stage in CRC [70, 163, 164, 167]. The overexpression of circRASSF2, circ_0082182, circ5615, circcct3, circ_0005075, and circRNA_100290 indicates a poor prognosis in CRC patients [70, 162, 163, 165, 167, 168]. Circ-PRKDC is also associated with lymph node metastasis and tumor size [164]. Circ_0005075 expression is correlated with differentiation and the depth of tumor invasion [162, 167]. Functionally, the expression of cis-HOX facilitates the self-renewal of colorectal tumor-initiating cells [201]. Circ-ABCC1 could regulate malignant phenotypes, such as cell sphere formation ability, cell migration, and cell stemness, in CRC [204]. The role of circ-PRKDC in 5-fluorouracil resistance has been reported [164]. Additionally, the other Wnt pathway-associated upregulated circRNAs (Table 1) inhibit CRC cell growth and metastasis [70, 202, 205–207].

#### Liver cancer

Hepatocellular carcinoma (HCC) is the most common type of primary liver cancer [235–238]. The expression of circRNA-SORE, circβ-catenin, circZFR, and circ_0067934 is relatively elevated in HCC [71, 172, 173, 210]. In particular, increased circRNA-SORE levels were found in sorafenib-resistant HCC. CircZFR and circ_0067934 levels are significantly associated with the prognosis of patients with HCC [172, 173]. The expression of circ_0067934 is also markedly correlated with tumor TNM stage in HCC [173]. CircRNA-SORE, cZNF292, circβ-catenin, circZFR, and circ_0067934 markedly facilitates cell proliferation [172, 173, 209, 210, 239]. Additionally, cZNF292, circRNA-SORE, and circ_0067934 reduce cell apoptosis [71, 173, 210, 239], while cZNF292 has no apparent effect on apoptosis [209]. The overexpression of circβ-catenin, circZFR and circ_0067934 increased the migration or invasion of HCC cancer cells [172, 173, 210]. High circRNA-SORE levels are important for maintaining HCC sorafenib resistance [71]. Mechanistically, some circRNAs interact with Wnt/β-catenin via other molecules in HCC. Circ_0067934 regulates HCC cell behaviors by activating the miR-1324/FZD5/wnt/β-catenin axis [173]. cZNF292 increases Wnt/β-catenin pathway activity through the upregulation of sex-determining region Y (SRY)-box 9 (SOX9) nuclear translocation [209].

On the other hand, the expression of circ_0004018, circ_0003418, and circ-ITCH is significantly downregulated in HCC [169, 170, 174, 208]. CircZKSCAN1 and circ-ITCH are potential prognostic biomarkers [171, 174]. Circ_0004018 and circ_0003418 are negatively correlated with tumor size [169, 170]. In addition, the expression of circ_0003418 has been reported to be related to TNM stage and HBsAg levels in HCC [170]. Circ_0004018 and circ_0003418 contribute to cancer development and progression by regulating many cell biological functions, including cell proliferation, migration, and invasion. Knockdown of circZKSCAN1 could inhibit the malignant behaviors of HCC cancer stem cells, such as sphere formation, colony formation, cell proliferation, and metastasis. Circ_0004018 modulates the Wnt/β-catenin pathway to accelerate HCC progression by targeting the miR-626/DKK3 axis. CircZKSCAN1 binds with FMRP to increase Wnt signaling activity in HCC.

#### Pancreatic cancer

Pancreatic cancer is a digestive tract malignancy with limited treatment options and poor life expectancy [240–243]. Pancreatic ductal adenocarcinoma is the most common primary malignancy of the pancreas [244–246]. The expression of circ_0030167 is significantly elevated in bone marrow mesenchymal stem cells (BM-MSCs) [211]. Yao et al. isolated BM-MSCs from human bone marrow. circ_0030167, obtained from BM-MSC-derived exosomes, attenuates pancreatic cancer cell growth, metastasis, and stemness. Exosomal circ_0030167 activates the WIF1/Wnt8/β-catenin axis by sponging miR-338-5p in pancreatic cancer. An increasing number of Wnt pathway-associated circRNAs have also been found in pancreatic ductal adenocarcinoma [247]. However, the underlying functions and mechanisms still need to be further explored.

### The respiratory system tumor

#### Lung cancer

**Fig. 4:**
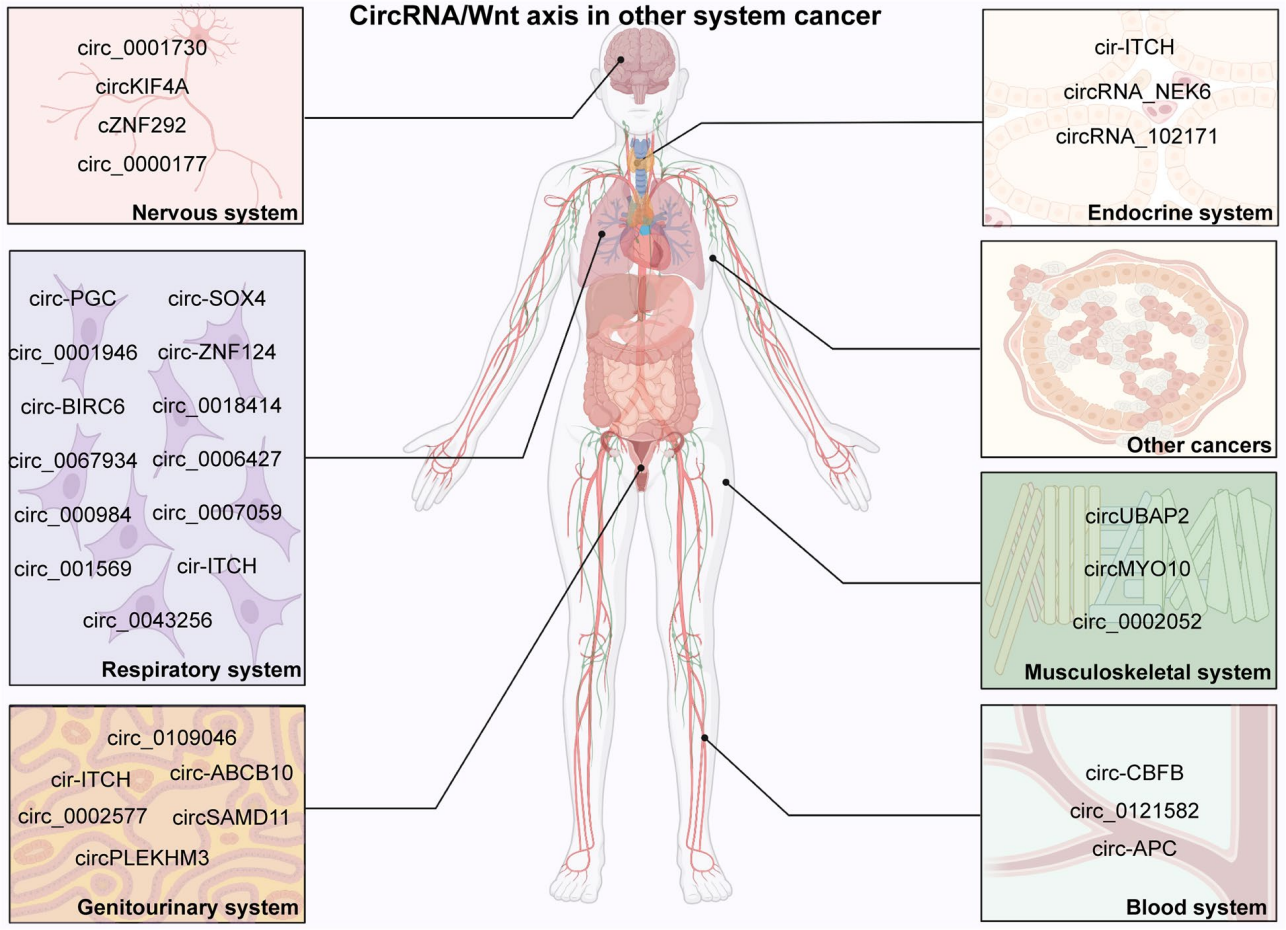
Wnt pathway-associated circRNAs in respiratory system cancer, nervous system cancer, genitourinary tumors, blood system cancers, musculoskeletal system cancer, endocrine system cancer, and cancers of other systems

Lung cancer is the main cause of cancer-associated mortality worldwide [248–252]. It can be classified into non-small-cell lung cancer (NSCLC) and small-cell lung cancer, and NSCLC accounts for the overwhelming majority of lung cancer cases [253–255]. Wnt pathway-associated circRNAs of NSCLC are shown in Table 1 [69, 175–181, 212–216] (Fig. 4). The overexpression of circ_000984 and circ_001569 is significantly correlated with TNM stage and lymph node metastasis in NSCLC [175, 176]. The circ_0001946 expression profile is obviously associated with TNM stage and tumor size in NSCLC [177]. Additionally, circ_000984, circ_001569, and circ_0001946 upregulation predicts a poor prognosis in patients with NSCLC [175–177]. These upregulated circRNAs in NSCLC could promote cell growth by enhancing cell proliferation [69, 175–177, 212–216]. *In vitro* astray assays showed that silencing circ_0067934 and circ_000984 could inhibit the epithelial-mesenchymal transition (EMT) process to reduce cell metastasis in NSCLC [175, 215]. Circ-PGC could also hinder cancer progression by suppressing glycolysis metabolism [212]. Mechanistically, the majority of circRNAs interact with miRNAs to activate the Wnt/β-catenin pathway in NSCLC [69, 177, 212–216].

**Fig. 5:**
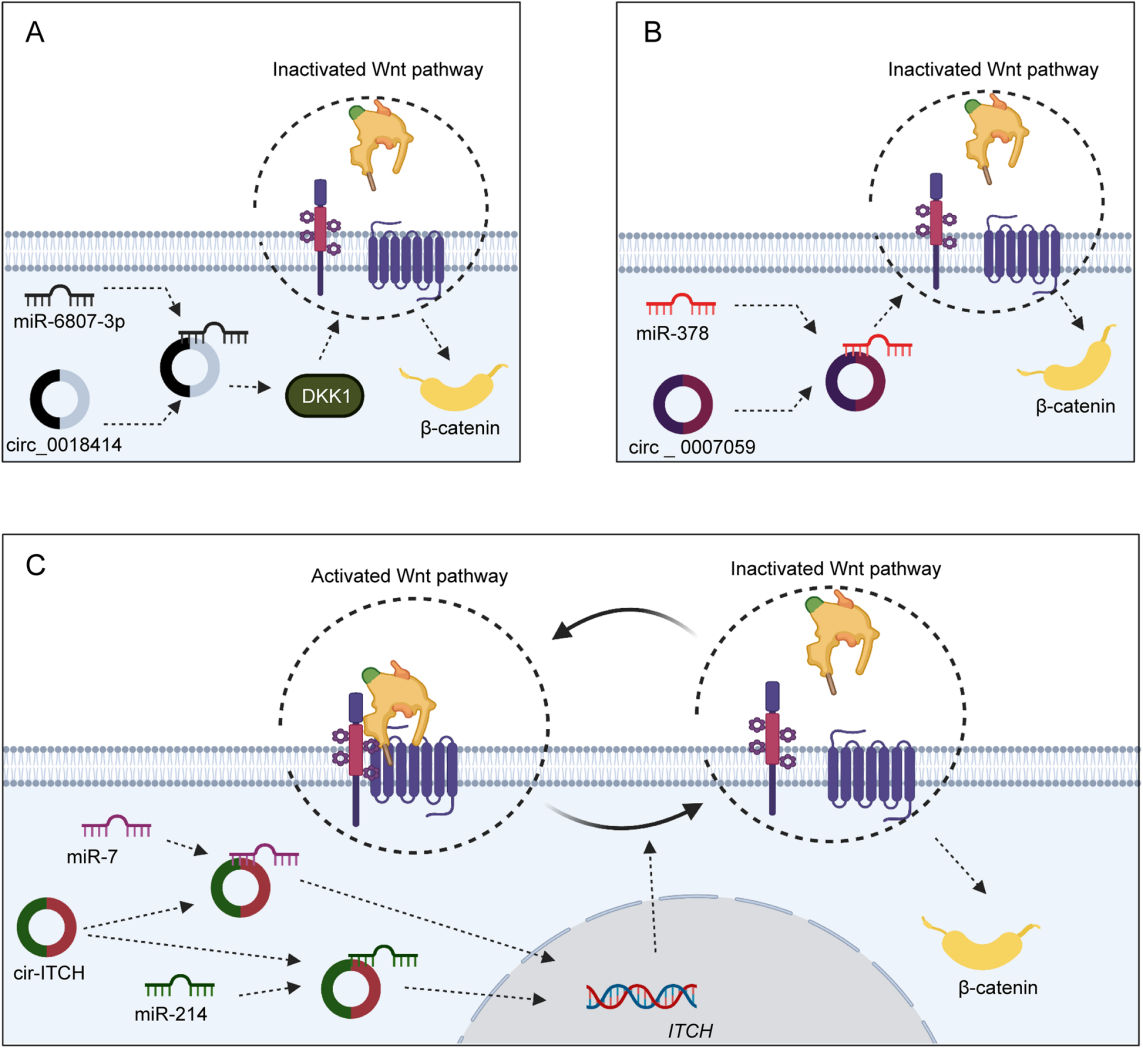
The mechanisms of Wnt-associated circRNA in lung cancer. **A** In lung cancer, circ_0018414 inhibits cancer progression by regulating miR-6807-3p/DKK1 and the Wnt/β-catenin pathway. DKK1 is upregulated by circ_0018414, a ceRNA of miR-6783-3p. **B** Circ 0007059 inhibits the Wnt/β-catenin by acting as a sponge for miR-378. **C** Cir-ITCH upregulates ITCH expression to inactivate Wnt/β-catenin signaling by sponging miR-7 and miR-214

Interestingly, circ_0018414, circ_0006427, circ_0007059, and cir-ITCH are remarkably downregulated in NSCLC [178–181]. Circ_0018414 and circ_0006427 are markedly associated with the overall survival rate [178, 179]. Circ_0006427 and circ_0007059 facilitate cell growth and motility in NSCLC [179, 180]. Circ_0018414 enhances stemness features by promoting DKK1 expression in NSCLC [178] (Fig. 5). CircRNAs can inhibit NSCLC tumorigenesis and progression by regulating the circ_0018414/miR-6807-3p/dkk1/Wnt/β-catenin, circ_0006427/ miR-6783-3p/dkk1/Wnt/β-catenin, and circ_0007059/miR-378/Wnt/β-catenin pathways and the cir-ITCH/miR-7/miR-214/ITCH/Wnt/β-catenin axis.

### Nervous system neoplasms

#### Glioma

Malignant gliomas are the most common primary tumors of the central nervous system [256–259]. Wnt pathway-associated circRNAs have drawn much attention in glioma research in recent years [260–263]. The levels of circ_0001730, circKIF4A, circ_0000177, and cZNF292 are upregulated in glioma [182, 183, 217, 218, 264] tissues versus normal brain tissues. Circ_0000177 is related to clinical stage, and patients with increased circ_0000177 expression have a poor prognosis [183]. Circ_0001730, circKIF4A, and circ_0000177 are all involved in tumor cell growth and metastasis in glioma [182, 183, 217]. cZNF292 promotes cancer development by regulating cell proliferation, the cell cycle, and angiogenesis. Mechanistically, circ_0001730 functions as a sponge of miR-326 to positively regulate Wnt/β-catenin pathways in the pathophysiologic processes of glioma. Circ_0001730 could also be upregulated by SP1 [218]. Overexpression of circ_0000177 increases FZD7 levels to activate Wnt signaling mediated by miR-638 in glioma.

### Genitourinary tumors

#### Prostate cancer (PCa)

PCa refers to an epithelial malignancy that occurs in the prostate [265–269]. The expression of cir-ITCH was significantly downregulated in PCa tissues and cell lines [219]. Further experiments showed that cir-ITCH could attenuate PCa cell viability and invasion. Cir-ITCH hinders PCa development by inactivating the Wnt/β-Catenin and PI3K/AKT/mTOR pathways. Not much is known about Wnt pathway-associated circRNAs in PCa. There is a crucial need for Wnt pathway-associated circRNA research in PCa [219].

#### Female reproductive system cancers

Cancers that originate in the female reproductive system are called female reproductive cancers [270]. Ovarian cancer (OC), endometrial cancer (EC), and cervical cancer are the three most common gynecological malignancies [271–274]. The expression of circ‑ABCB10 is significantly upregulated, while circPLEKHM3 expression is downregulated in OC [184, 220]. Moreover, the level of circPLEKHM3 is positively associated with the overall survival rate in patients with OC [184]. Circ‑ABCB10 remarkably facilitates cell proliferation and invasion and reduces cell apoptosis by miR-1271 in OC [220]. Circ‑ABCB10 plays a critical role in OC progression via the regulation of Capn4/Wnt/β‑catenin. CircPLEKHM3 inhibits cell proliferation and migration by sponging miR-9 and regulating the BRCA1/DNAJB6/KLF4/AKT1/Wnt/β-catenin axis in OC [184]. Circ_0109046 and circ_0002577 are elevated in EC tissues and cell lines [185, 186]. The overexpression of circ_0002577 is positively correlated with advanced FIGO stage and lymph node metastasis in EC. High expression of circ_0109046 and circ_0002577 predicts a poor prognosis in patients with EC. Circ_0109046 activates the Wnt/β-catenin pathway by sponging miR-105 to increase SOX9 levels. Circ_0002577 functions as a sponge of miR-197 to regulate the CTNND1/Wnt/β-catenin axis in EC. CircSAMD11 expression is markedly upregulated in cervical cancer [221]. Silencing of circSAMD11 expression suppressed cell proliferation and metastasis and promoted cell apoptosis in cervical cancer. The circSAMD11/miR-503/sox4/Wnt/β-catenin axis plays an essential role in the progression of cervical cancer [221].

## Tumors of the blood system

Hematological malignancies, also known as neoplasms of the blood, lymph nodes and bone marrow, include leukemia, lymphoma, and multiple myeloma [275–278]. The common types of leukemia are acute myeloid leukemia (AML), chronic myeloid leukemia (CML), acute lymphoblastic leukemia (ALL), and chronic lymphocytic leukemia (CLL) [279–282]. Circ_0121582 expression is significantly decreased in AML [222]. Functional experiments demonstrated that the overexpression of circ_0121582 significantly attenuated cell survival and promoted the cell cycle arrest in AML. Circ_0121582 activates Wnt/β-catenin by sponging miR-224 to increase GSK3β expression in AML. The expression of circ-CBFB was upregulated in CLL, and it has been reported as an independent predictive factor for the prognosis of CLL [223]. Circ-CBFB facilitates CLL cell proliferation and inhibits cell apoptosis by sponging miR-607 and upregulating the FZD3/Wnt/β-catenin axis. Diffuse large B-cell lymphoma (DLBCL) is the most common malignant lymphoma subtype [283–285]. The level of circ-APC is significantly decreased in the tissues, cell lines, and plasma of DLBCL patients versus normal controls [224]. circ-APC inactivates the Wnt/β-catenin pathway to suppress cell proliferation in DLBCL through the regulation of the miR-888/APC and TET1/APC axes.

### Tumors of the musculoskeletal systems

#### Osteosarcoma (OS)

OS is the most common primary malignant neoplasm of the bone and mainly affects children, adolescents, and young adults [286–289]. The level of circMYO10 is significantly elevated [63], while circ_0002052 expression is downregulated in OS tissues and cell lines [187]. The expression of circ_0002052 is positively associated with overall and progression-free survival in patients with OS. circ_0002052 inhibits cell growth and cell motility and enhances cell apoptosis in OS by sponging miR-1205 and modulating the APC2/Wnt/β-catenin axis. CircMYO10 functions as an oncogene in OS progression. The overexpression of circMYO10 facilitates OS cell proliferation and EMT *in vitro*. CircMYO10 facilitates histone H4K16 acetylation by regulating the miR-370-3p/RUVBL1 axis and activating Wnt/β-catenin signaling in OS. Cisplatin (DDP) is a conventional chemotherapy drug in the treatment of OS [290–293]. Cisplatin resistance is a major challenge for OS chemotherapy application [294, 295]. CircUBAP2 expression is increased in cisplatin-resistant OS tissues and cells [225]. Silencing circUBAP2 inhibits cell proliferation, migration, and invasion and induced apoptosis in OS. CircUBAP2 knockdown also suppresses cisplatin resistance by regulating miR-506-3p/SEMA6D and the Wnt/β-catenin pathway [225].

### Tumors of the endocrine system

#### Thyroid cancer

The incidence rate of thyroid cancer has been increasing throughout the world [296–300]. CircRNA_102171 and circRNA_NEK6 are relatively upregulated [226, 227], while circ-ITCH is downregulated in thyroid cancer tissues and cell lines [188]. The level of circ-ITCH is closely associated with clinical stage, lymph node metastasis, and patient prognosis in thyroid cancer. CircRNA_102171 and circRNA_NEK6 play a promoting role in cell growth and metastasis. CircRNA_102171 activates the Wnt/β-catenin pathway in a CTNNBIP1-dependent way [226]. CircRNA_NEK6 facilitates thyroid cancer progression by sponging miR-370-3p and upregulating the FZD8/Wnt axis [227]. Circ-ITCH exerts its tumor suppressor action by modulating miR-22-3p/CBL/β-catenin in thyroid cancer [188].

### Tumors of other systems

Breast cancer is one of the most common malignant malignancies among females worldwide [301–304]. Circ-EIF6, circARL8B, circABCC4, circRNA_069718, and circFAT1 expression levels are obviously upregulated in breast cancer [189, 191, 228–230]. CircRNA_069718 overexpression is positively correlated with TNM stage, lymph node metastasis, and overall survival in patients with breast cancer [191]. These upregulated Wnt-associated circRNAs contribute to cancer progression by promoting cell growth and metastasis. In addition, studies also observed that knockdown of circARL8B could induce a suppressive effect on fatty acid metabolism in breast cancer [228]. CircFAT1 enhances oxaliplatin resistance through the miR-525-5p/SKA1 and Wnt pathways in breast cancer [230]. CircARL8B, circABCC4, and CircFAT1 regulate the Wnt pathway by acting as sponges of miRNAs in breast cancer. EIF6-224aa, encoded by circ-EIF6, activates Wnt/β-catenin by regulating the MYH9/Wnt/beta-catenin pathway [189].

Melanoma is a potentially fatal disease with increasing incidence [305–309]. Circ_0027247 was isolated from circ-GLI1 [232]. Circ_0119872, circ_0084043 and circ-GLI1 (circ_0027247) are dramatically upregulated in melanoma tissues and cell lines [231–233]. High levels of circ_0027247 and circ_0084043 can promote cell motility [232], while circ_0119872 has no influence on cell migration and invasion [231]. Circ_0119872 and circ_0027247 are novel negative feedback regulators of angiogenesis in melanoma. Circ_0119872 and circ_0084043 have the same effects on cell proliferation. Circ_0119872 activates the Wnt/β-catenin pathway by interacting with p70S6K2 and upregulates Cyr61 expression in melanoma. The tumorigenesis and progression of melanoma are also regulated by the circ_0119872/ p70S6K2/Wnt/β-catenin and circ_0027247/miR-622/G3BP1/Wnt/β-catenin axes [231].

### CircRNA, a potential biomarker in wnt pathway

Despite technological advances, cancer diagnosis and treatment are still a challenge that may require the emergence of new tumor biomarkers [310, 311]. Increasing evidence has revealed that Wnt-associated circRNAs are closely related to cancer progression. Wnt-associated circRNAs may be very promising biomarkers in cancer diagnosis, prognosis, and treatment. In this section, we will further discuss their potential application in clinical practice.

#### Diagnosis

The early screening and diagnosis of cancer is conducive to the survival of cancer patients [312–316]. Identifying suitable biomarkers has always been a difficult issue in cancer research. Wnt-associated circRNAs may be used to assist early diagnosis in many cancers. They are aberrantly expressed in many kinds of tumors from multiple systems, such as digestive tumors, respiratory system tumors, nervous system neoplasms, genitourinary tumors, musculoskeletal system tumors and endocrine system cancers. Moreover, plasma circ-APC levels are significantly downregulated in DLBCL [224]. This discovery indicates a more convenient clinical application of circ-APC as a diagnostic marker. Studies further evaluated the diagnostic potential for cancer by receiver operating characteristic (ROC) curve analysis. Yang et al. found that the AUC value of circ0005654 was 0.781 in gastric cancer [159]. ROC analysis of circRASSF2 expression levels in colorectal cancer tissues and cells accurately discriminated between CRC patients and healthy controls (AUC: 0.9863) [162]. Further experimental verification and research on circRASSF2 in body fluids is necessary. The corresponding AUC value for circ-CBFB was 0.80 in chronic lymphocytic leukemia [223].

#### Prognosis prediction

Early prognostic information is important in making treatment decisions [317–321]. A growing amount of evidence shows that Wnt-associated circRNAs can be of important prognostic value. These circRNAs are closely related to overall survival, disease-free survival, recurrence-free survival, 5-year survival rate, and progression-free survival in several cancers. Patients with lower circZKSCAN1 expression have shorter overall and recurrence-free survival in HCC [171]. Li et al. [166] reported that the overexpression of circCCT3 was negatively correlated with the disease-free survival rate in colorectal cancer. Higher circ_0109046 expression predicts a decreased 5-year survival rate in patients with endometrial carcinoma [185]. Such studies have important implications in prognosis evaluation and treatment selection. In addition, Wnt-associated circRNAs are associated with other relevant prognostic factors. For example, downregulated circMTO1 levels predict advanced TNM stage and lymph node metastasis in CRC [70].

#### Cancer treatment

Despite rapidly progressing treatment modalities, cancer therapy remains one of the most challenging issues in the world. CircRNA-based targeted therapeutic strategies shed new light on the evolution of cancer treatment [42, 43, 262, 322, 323]. CircRNAs regulate many cell biological functions by directly or indirectly interacting with the Wnt pathway. CircRNA_NEK6 activated the FZD8/Wnt axis to facilitate thyroid cancer progression by sponging miR-370-3p [227]. Circ_0121582 promotes GSK3β expression to activate the Wnt/β-catenin pathway by sponging miR-224 in AML [222]. Circ-SFMBT2 contributes to the development and tumorigenesis of gastric cancer via regulation of the miR-1276/CTNNB1/Wnt/β-catenin axis [195]. Controlling Wnt-associated circRNA expression may be an effective approach for cancer treatment. The knockdown of circ_SMAD4 blocked gastric cancer progression by negatively regulating cell growth [73]. Silencing circ-ZNF124 expression inhibited malignant phenotypes in NSCLC cells [73]. In addition, Circ-ITCH is a tumor suppressor in many cancers [160, 174, 181, 188, 190, 193, 207, 208, 219]. Wang et al. found that upregulated circ-ITCH expression suppressed cell proliferation and invasion in papillary thyroid cancer [188]. However, the identification of targeted drugs that can stably control the expression of circRNA and transmit this effect is the current difficulty. This requires a deeper understanding of the structure and function of Wnt-associated circRNAs. The majority of circRNAs act as sponges of miRNAs to activate or inactivate the Wnt pathway. Regulating the target miRNAs of Wnt-associated circRNAs may also be feasible. MiR-582 intervention effectively reversed the cell biological functions regulated by circ_0009361 in CRC [205].

## Conclusions and future perspectives

The Wnt signaling pathway is highly involved in cancer development, and essential for a wide variety of cellular functions, such as cell polarity, movement, proliferation, asymmetric division, and muscle tissue development. Both circRNA and the Wnt pathway play a critical role in cancer development and progression. Emerging data suggest that the circRNA/Wnt axis modulates the expression of cancer-associated genes and then regulates tumor progression. CircRNAs are enriched in the Wnt pathway. Wnt-associated circRNAs are abnormally expressed in digestive tumors, respiratory system tumors, nervous system neoplasms, genitourinary tumors, musculoskeletal system tumors, endocrine system cancers and other cancers. Their aberrant expression indicates their potential as diagnostic markers. However, most related experiments are based on tissue and cell research. Ideal and effective molecular markers should be stably expressed in plasma, serum, and other body fluids. Such molecules have greater potential for clinical applications. Wnt-associated circRNAs are also promising potential biomarkers in the treatment of cancer. CircRNAs negatively or positively regulate cancer initiation, promotion, and progression by directly or indirectly interacting with the Wnt pathway. We could enhance the expression of cancer-promoting circRNAs or inhibit the expression of tumor suppressor circRNAs to control cancer progression. The current goal is to find targeted drugs that can stably control the expression of circRNA and induce this effect. We need to further understand the structure and function of Wnt-related circRNAs. Furthermore, the interaction and the related mechanisms between circRNAs involved in the Wnt pathway need more studies to confirm.

## Data Availability

Not applicable.

## Abbreviations

ncRNAs: noncoding RNAs
miRNAs: microRNAs
lncRNAs: long ncRNAs
circRNAs: circular RNAs
PCP: Wnt/planar cell polarity
GSK-3β: glycogen synthase kinase 3β
TCF: T cell transcription factor
LEF: lymphoid enhancer factor
ceRNAs: competing endogenous RNAs
ESCC: esophageal squamous cell carcinoma
CCK-8: Cell Counting Kit-8
CRC: colorectal cancer
TNM: tumor-node-metastasis
HCC: hepatocellular carcinoma
SRY: sex-determining region Y
SOX9: sex-determining region Y-box 9
BM-MSCs: bone marrow mesenchymal stem cells
NSCLC: non-small-cell lung cancer
EMT: epithelial-mesenchymal transition
KPS: Karnofsky Performance Status
OC: ovarian cancer
EC: endometrial cancer
AML: acute myeloid leukemia
CML: chronic myeloid leukemia
ALL: acute lymphoblastic leukemia
CLL: chronic lymphocytic leukemia
DLBCL: diffuse large B-cell lymphoma
OS: osteosarcoma
DDP: cisplatin
ROC: receiver operating characteristic
